# Prevalence of an angiotensin-converting enzyme gene variant in dogs

**DOI:** 10.1186/s40575-021-00105-2

**Published:** 2021-07-13

**Authors:** D. B. Adin, C. E. Atkins, S. G. Friedenberg, J. A. Stern, K. M. Meurs

**Affiliations:** 1 University of Florida, College of Veterinary Medicine, Gainesville, FL USA; 2 North Carolina State University, College of Veterinary Medicine, Raleigh, NC USA; 3 University of Minnesota, College of Veterinary Medicine, Saint Paul, MN USA; 4 University of California, Davis, Davis, CA USA

**Keywords:** Renin-angiotensin aldosterone system, Heart, Genotype, Aldosterone breakthrough

## Abstract

**Background:**

Genetic heterogeneity of the canine angiotensin converting enzyme (ACE) gene is functionally important because the degree of aldosterone breakthrough with ACE-inhibitor therapy is greater in variant positive dogs compared to variant negative dogs, but the prevalence of the variant is not known. The purpose of this study was to determine ACE gene variant-positive prevalence in a population of 497 dogs of different breeds.

**Results:**

Overall variant-positive prevalence was 31%, with 20% of dogs heterozygous and 11% of dogs homozygous. The variant was overrepresented in Irish Wolfhounds (prevalence 95%; *P* < .001), Dachshunds (prevalence 90%; *P* < .001), Cavalier King Charles Spaniels (prevalence 85%; *P* < .001), Great Danes (prevalence 84%; *P* < .001), and Bull Mastiffs (prevalence 58%; *P* = .02). Irish Wolfhounds were more likely to be homozygous than heterozygous (*P* < .001).

**Conclusions:**

Nearly one-third of dogs in this study were positive for a functionally important ACE gene variant, with wide prevalence variability between breeds. The clinical importance of high ACE gene variant-positive prevalence in some breeds requires further study because the highest prevalences were found in breeds that are predisposed to heart disease and therefore may be treated with ACE-inhibitors.

## Plain English summary

Genetic differences in the angiotensin converting enzyme (ACE) have been shown to affect the way dogs respond to ACE-inhibitors, a class of medication which is used to treat dogs with heart disease. Dogs that have a genetic mutation of the ACE gene show less benefit with ACE-inhibitor medications than dogs without the mutation, but it is not known how common the mutation is in dogs. The purpose of this study was to determine the prevalence of this mutation (variant) in a large population of dogs of different breeds. The overall variant-positive prevalence in this population of 497 dogs was 31%. The variant was overrepresented in Irish Wolfhounds (prevalence 95%), Dachshunds (prevalence 90%), Cavalier King Charles Spaniels (prevalence 85%), Great Danes (prevalence 84%), and Bull Mastiffs (prevalence 58%). Irish Wolfhounds were more likely than other breeds to have 2 copies of the mutation (homozygous) than 1 copy of the mutation (heterozygous). The clinical importance of high ACE gene variant-positive prevalence in some breeds will require additional studies because some breeds are predisposed to heart disease, for which treatment with ACE-inhibitor medication might be recommended.

## Background

The angiotensin converting enzyme (ACE) is an important catalyst for the renin-angiotensin aldosterone system (RAAS) cascade by cleaving a carboxy-terminal dipeptide from angiotensin I to form angiotensin II [1]. When the RAAS is pathologically and chronically activated, angiotensin II, both directly and through stimulation of aldosterone synthesis, mediates maladaptive actions such as vasoconstriction, sodium retention, and pathological remodeling of cardiovascular and renal tissues [1]. Inhibitors of ACE are used clinically to reduce the formation of angiotensin II, thereby mitigating these effects which contribute to cardiovascular and renal disease progression, fluid retention, and systemic hypertension [1, 2]. Clinically effective RAAS suppression by ACE-inhibitors, however, is sometimes sub-optimal because of aldosterone breakthrough, non-ACE mediated angiotensin II formation, genetic variants affecting RAAS components, feedback mechanisms, and likely other unidentified factors [3–7]. The ACE gene intronic variant at canine chromosome 9:11507816:G > A has recently been shown to increase the magnitude of aldosterone breakthrough, despite adequate suppression of angiotensin II by ACE-inhibitors [4]. Genotype influence on non-angiotensin mediated aldosterone production holds clinical implications for dogs with advanced heart disease in both disease expression and response to treatments, but the prevalence of this variant in the canine population is unknown. This study sought to determine the prevalence of this ACE gene variant in a large number of dogs and to define breed predispositions. We hypothesized that this ACE gene variant would be common in dogs, but with variable breed distributions.

## Results

The variant was present in 32 breeds and absent in 22 breeds in the 497 dogs screened (Table 1). Allele frequency of the chromosome 9:11507816:G > A variant within the population was 21%. Overall variant-positive prevalence within the study population was 31%, with 20% of dogs heterozygous, and 11% of dogs homozygous.

**Table 1:** The number and genotype of 497 dogs for each breed represented in this study is shown

Breed	Number of Dogs	Wild type	Heterozygous	Homozygous
**American Foxhound**	1	0	1	0
**American Staffordshire Terrier**	1	0	1	0
**Australian Shepherd**	3	1	2	0
**Bichon Frisé**	3	3	0	0
**Border Collie**	8	5	1	2
**Boston Terrier**	1	1	0	0
**Bouvier**	10	7	3	0
**Boxer**	20	19	1	0
**Boykin Spaniel**	1	1	0	0
**Bull Mastiff**	19	8	10	1
**Bulldog**	28	21	6	1
**Cairn Terrier**	7	3	3	1
**Cavalier King Charles Spaniel**	13	2	7	4
**Collie**	4	4	0	0
**Coonhound**	1	1	0	0
**Corgi**	8	3	4	1
**Dachshund**	10	1	3	6
**Doberman Pinscher**	5	1	2	2
**English Bulldog**	3	3	0	0
**English Cocker Spaniel**	1	1	0	0
**English Mastiff**	1	1	0	0
**French Bulldog**	18	18	0	0
**German Shepherd**	2	1	1	0
**Golden Retriever**	42	38	4	0
**Goldendoodle**	2	1	1	0
**Great Dane**	19	3	8	8
**Great Pyrenees**	3	3	0	0
**Havanese**	2	0	1	1
**Irish Setter**	3	1	1	1
**Irish Wolfhound**	20	1	3	16
**Labradoodle**	2	2	0	0
**Labrador Retriever**	12	6	6	0
**Lhasa Apso**	3	3	0	0
**Miniature Poodle**	10	6	2	2
**Miniature Schnauzer**	19	19	0	0
**Mixed breed**	7	7	0	0
**Newfoundland**	15	14	1	0
**Pomeranian**	13	13	0	0
**Portuguese Water Dog**	2	2	0	0
**Pug**	3	2	1	0
**Rhodesian Ridgeback**	3	0	0	3
**Rottweiler**	16	15	1	0
**Scottish Deerhound**	10	7	2	1
**Scottish Terrier**	6	6	0	0
**Sheltie**	9	9	0	0
**Shih Tzu**	1	1	0	0
**Siberian Husky**	12	8	4	0
**Standard Poodle**	33	28	2	3
**Toy Poodle**	4	2	2	0
**Welsh Springer Spaniel**	4	4	0	0
**Welsh Terrier**	1	1	0	0
**West Highland White Terrier**	4	4	0	0
**Whippet**	15	9	6	0
**Yorkshire Terrier**	34	24	9	1

Twenty-one breeds were represented by ≥10 dogs and were evaluated for breed predisposition for the variant (Table 2). The variant was overrepresented in Irish Wolfhounds (prevalence 95%; *P* < .001), Dachshunds (prevalence 90%; *P* < .001), Cavalier King Charles Spaniels (prevalence 85%; *P* < .001), Great Danes (prevalence 84%; *P* < .001), and Bull Mastiffs (prevalence 58%; *P* = .02). The variant was underrepresented in Golden Retrievers (prevalence 10%; *P* = .001), Newfoundlands (prevalence (7%; *P* = .046), Rottweilers (prevalence 6%; *P* = .03), Boxers (prevalence 5%; *P* = .01), Miniature Schnauzers (prevalence 9%; *P* = .001), French Bulldogs (prevalence 0%; *P* = .001), and Pomeranians (prevalence 0%; *P* = .01). Of the 11 breeds with ≥5 variant-positive dogs, only Irish Wolfhounds were more likely to be homozygous than heterozygous (*P* < .001; Table 3).

**Table 2:** Breed predispositions for genotype are shown for breeds with ≥10 dogs

Breed	n	VP (n, %)	VN (n, %)	***P*** value	Odds ratio	95% CI
Bouvier	10	3 (30)	7 (70)	1.0	0.963	0.268 to 3.493
Boxer	20	1 (5)	19 (95)	**.01**	0.113	0.011 to 0.633
Bull mastiff	19	11 (58)	8 (42)	**.02**	3.254	1.317 to 7.994
Bulldog	28	7 (25)	21 (75)	.67	0.737	0.289 to 1.738
Cavalier King Charles Spaniel	13	11 (85)	2 (15)	**<.001**	13.250	3.187 to 60.260
Dachshund	10	9 (90)	1 (10)	**<.001**	21.440	3.493 to 236.0
French Bulldog	10	0 (0)	10 (100)	**.04**	0.000	0.000 to 0.426
Golden Retriever	42	4 (10)	38 (90)	**.001**	0.216	0.081 to 0.584
Great Dane	19	16 (84)	3 (16)	**<.001**	13.270	4.112 to 43.370
Irish Wolfhound	21	19 (90)	2 (10)	**<.001**	48.630	8.598 to 509.700
Labrador Retriever	12	6 (50)	6 (50)	.20	2.299	0.714 to 7.376
Miniature poodle	10	4 (40)	6 (60)	.51	1.512	0.475 to 5.683
Miniature Schnauzer	19	0 (0)	19 (100)	**.001**	0.000	0.000 to 0.398
Newfoundland	15	1 (7)	14 (93)	**.046**	0.155	0.015 to 0.943
Pomeranian	13	0 (0)	13 (100)	**.012**	0.000	0.000 to 0.6424
Rottweiler	16	1 (6)	15 (94)	**.029**	0.144	0.0136 to 0.860
Scottish Deerhound	10	3 (30)	7 (70)	1.0	0.963	0.268 to 3.493
Siberian Husky	12	4 (33)	8 (67)	1.0	1.128	0.372 to 3.962
Standard Poodle	33	5 (15)	28 (85)	.05	0.381	0.157 to 0.969
Whippet	15	6 (40)	9 (60)	.41	1.519	0.534 to 4.089
Yorkshire Terrier	34	10 (29)	24 (71)	1.0	0.932	0.455 to 1.978

Odds ratio and 95% CI > 1.0 indicated overrepresentation while < 1.0 indicated underrepresentation. *P* < .05 bolded. *VN* variant-negative (wild type), *VP* variant-positive (heterozygous or homozygous), *n* number of dogs in each breed

**Table 3:** The homozygosity odds ratios for breeds with ≥5 variant-positive dogs are shown

Breed	n (VP)	Homozygous (#, %)	Heterozygous (#, %)	***P*** value	Odds ratio	95% CI
Bull Mastiff	11	1 (9)	10 (91)	.10	0.183	0.0166 to 1.130
Bulldog	7	1 (14)	6 (86)	.42	0.306	0.0262 to 1.962
Cavalier King Charles Spaniel	11	4 (36)	7 (64)	1.0	1.048	0.331 to 3.510
Corgi	5	1 (20)	4 (80)	.66	0.458	0.0368 to 2.891
Dachshund	9	6 (67)	3 (33)	.08	3.667	0.964 to 13.690
Great Dane	16	8 (50)	8 (50)	.28	1.833	0.637 to 5.255
Irish Wolfhound	19	16 (84)	3 (16)	**<.001**	9.778	2.816 to 32.410
Labrador Retriever	6	0 (0)	6 (100)	.10	0.000	0.000 to 1.371
Standard Poodle	5	3 (60)	2 (40)	.35	2.750	0.544 to 15.760
Whippet	6	0 (0)	6 (100)	.10	0.000	0.000 to 1.371
Yorkshire Terrier	10	1 (10)	9 (90)	.17	0.204	0.0183 to 1.316

Odds ratio and 95% CI > 1.0 indicated overrepresentation while < 1.0 indicated underrepresentation. *P* < .05 bolded. *VP* variant-positive, *n* number of dogs in each breed

## Discussion

The ACE gene variant at chromosome 9:11507816:G > A was found in nearly one-third of dogs, with unequal breed distribution. The breeds with the highest prevalences include some that are predisposed to heart disease, which could have clinical importance if RAAS suppression is indicated for treatment [4]. Two of the breeds with high variant-positive prevalence (≥84%) are predisposed to dilated cardiomyopathy (Irish Wolfhound and Great Dane) and 2 other breeds with high variant-positive prevalence (≥85%) are predisposed to degenerative mitral valve disease (Cavalier King Charles Spaniel and Dachshund) [8–11]. Both of these cardiac diseases are commonly treated with ACE-inhibitors. Our study also showed that Irish Wolfhounds were more likely to be homozygous for the variant than heterozygous, but the clinical importance of this finding is unknown. Conversely, 7 other breeds were less likely to have the ACE gene variant and some of these breeds are also predisposed to cardiac disease (e.g. Boxer, Pomeranian, Miniature Schnauzer).

The efficacy of ACE-inhibitors in delaying disease progression and prolonging survival in dogs with both preclinical and clinical mitral valve disease is debated because published study results are contradictory [12–15]. The relatively high prevalence of a functionally important ACE gene variant in some breeds but not others and the effect of this variant on aldosterone breakthrough, could explain discordant findings depending on the enrolled population of dogs [4]. Future studies investigating the efficacy of ACE-inhibitors in dogs with naturally occurring disease should consider genotype in study planning.

This study has limitations that impact interpretation. Although the database was relatively large, the number of dogs in each breed varied and was low for some breeds. Therefore, breed-specific assessments may have been underpowered and findings could be different with a greater number of dogs. Additionally, we did not analyze for breed predisposition if the number of dogs in the database was < 10 for a breed, and not all breeds were represented in the database. Therefore, other breed predispositions may be present but not uncovered by this study. Although the database did not include large families, the degree of relatedness between dogs was not explored in this study. Specific geographic origins within North America were not available and so the potential for geographic genotypic differences in the ACE gene variant remains unexplored. It is also possible that variant-positive prevalence was linked to the disease for which the dogs were sequenced, and therefore prevalence within larger or different populations could be different.

## Conclusions

Nearly one-third of dogs in this study were positive for a functionally important ACE gene variant, and for some breeds predisposed to naturally occurring heart disease, the prevalence was much higher. Genotyping for this variant may advance personalized canine medicine and permit targeted clinical trials in the future.

## Methods

A database of 497 canine whole genome sequences from 54 breeds sequenced for various disease-specific studies from 10/1/2014 to 12/31/2020 was utilized to genotype dogs at chromosome 9:11507816. All sequenced dogs were from North America but the specific geographic origins were not available. The reasons for sequencing in this group of dogs were: cardiac (57.1%), neurologic (8.5%), renal (8.2%), immunologic (5.8%), musculoskeletal (6.2%), respiratory (2.6%), ophthalmologic (2.6%), healthy (3.4%), unspecified (1.8%), hepatobiliary (1.6%), metabolic (1.6%), and dermatologic (0.4%).

Samples were classified as wild type, heterozygous, or homozygous for the ACE gene variant at chromosome 9:11507816:G > A. Allele frequency and overall variant-positive prevalence (heterozygous and homozygous) within our canine study population were calculated.

Breed predisposition was evaluated for breeds with ≥10 dogs in the dataset by using Fisher’s exact test to compare the variant-positive prevalence of each breed to that of all the remaining dogs in the sample population. Effect size was determined by calculating the odds ratio for each breed (the odds of being variant-positive for each of these breeds was divided by the odds of being variant-positive for all the other dogs in the sample population except those of that particular breed). Breeds were considered overrepresented if the odds ratio (and 95% CI) of having the variant was > 1.0 and underrepresented if < 1.0.

Differences in homozygous and heterozygous status were evaluated for breeds with ≥5 variant-positive dogs using Fisher’s exact test to compare the prevalence of homozygosity for each of these breeds to the prevalence of homozygosity for variant-positive dogs from the other breeds combined. Breeds without variant-positive dogs (i.e. breeds with only wildtype dogs) were not used for this analysis. Effect size was determined by calculating the odds ratio for each breed (the odds of being homozygous for each of these breeds divided by the odds of being homozygous for the variant-positive dogs from all the other breeds combined. Homozygosity was considered overrepresented in a breed if the odds ratio (and 95% CI) of being homozygous was > 1.0 and underrepresented if < 1.0. Statistical analysis was performed using commercially available software (GraphPad Prism 8, San Diego CA, USA). Significance was set at *P* < .05.

## Data Availability

Datasets are presented within the manuscript. Data for individual dog genotyping are available from the corresponding author upon reasonable request.

## Abbreviations

ACE: Angiotensin converting enzyme
RAAS: Renin-angiotensin-aldosterone system
